# Using Geometric Morphometric Analysis of Magnetic Resonance Imaging to Assess the Anatomy of the Eustachian Tube in Children with and without Otitis Media

**DOI:** 10.3390/bioengineering10101115

**Published:** 2023-09-23

**Authors:** Ellen E. Fricano, Allison P. Gremba, Miriam S. Teixeira, J. Douglas Swarts, Cuneyt M. Alper

**Affiliations:** 1 College of Osteopathic Medicine of the Pacific, Western University of Health Sciences, Pomona, CA 91766, USA; 2 Doctor of Physical Therapy Program, School of Natural and Health Sciences, Seton Hill University, Greensburg, PA 15601, USA; 3 Department of Graduate Medical Education, Arnot Ogden Medical Center, Elmira, NY 14905, USA; 4 Department of Otolaryngology, University of Pittsburgh School of Medicine, Pittsburgh, PA 15213, USA; 5 Division of Pediatric Otolaryngology, University of Pittsburgh Medical Center Children’s Hospital of Pittsburgh, Pittsburgh, PA 15224, USA

**Keywords:** Eustachian, pharyngotympanic, craniofacial growth, biomedical imaging, MRI scans, geometric morphometric, Procrustes analysis, canonical variate analysis, principal component analysis

## Abstract

Otitis media (OM) is among the most common of childhood illnesses. It has long been hypothesized that children under age two are predisposed to OM due to differences in the anatomy of the Eustachian tube (ET), including the angle of the ET. OM in later childhood is less common but does occur, begging the question, are there shape differences in the ET that persist underlying later occurrences of OM? To answer this question, a novel method, which applied geometric and morphometric shape analysis to landmarks obtained from MRI data, was used. MRI scans were performed on 16 children (5 control, 3 cOME, and 8 rAOM) between 2011 and 2015. Sixteen landmarks representing the shape of the ET, cranial base, and palate were analyzed. The results of a Procrustes ANOVA indicate that the shape of the ET varies significantly (*p* < 0.01) between the OM and control groups. The shape differences between the OM group and the control are a medial and low attachment site of the tensor veli palatini (TVP) muscle, a posterior and high torus tubarius, and an anteriorly projected palate. These results support previous findings that a relatively horizontal ET is associated with a predisposition for OM. This study used a novel approach to examine anatomical differences in children with and without OM. First, the data set is unique in that it includes MRI scans of children with a confirmed OM diagnosis. Second, the use of MRI scans in craniofacial anatomy OM research is novel and allows for the collection of soft tissue landmarks and the visualization of soft tissue structures. Third, geometric morphometric shape analysis is a statistical method that captures shape differences, offering a more universal picture of nuanced changes within the entire set of landmarks, in contrast to more traditional linear and angular measurements used in prior OM studies examining craniofacial anatomy.

## 1. Introduction

In the wake of a global pandemic, respiratory health and predisposition for upper respiratory tract infections are at the forefront of concerns for health professionals, patients, and parents. Upper respiratory tract infections are often complicated with coinciding middle ear infections in children [1,2,3]. There is a range of middle ear pathologies distinguished by frequency, duration, and fluid with and without signs of infection (e.g., otitis media with effusion (OME), acute otitis media (AOM), recurrent acute otitis media (rAOM), chronic otitis media with effusion (cOME), and chronic suppurative otitis media (CSOM)). Otitis media (OM) peaks in prevalence at approximately one to two years of age; the incidence decreases after age six, although cases can be seen into adolescence and adulthood [4,5,6,7,8]. This correlation between age, respiratory health, and OM has been linked to an immature immune system and several anatomical and functional differences, including the relative horizontal Eustachian tube (ET) in young children [9,10,11,12].

The ET’s function is several-fold: (1) pressure equalizing—when the lumen is dilated, air pressure in the middle ear can equalize with the external environment; (2) clearance—the ET provides a channel through which middle ear secretions and pathogens can drain; and (3) protection—the ET’s normally closed lumen prevents particulates, pathogens, nasopharyngeal sounds, and pressure variations from easily accessing the middle ear [13,14]. The ET lumen is closed at rest but can be opened passively by either middle ear or nasopharyngeal pressures that exceed that of the paratubal tissue or action of paratubal muscles, mainly the tensor veli palatini (TVP) and levator veli palatini muscles (LVP) [15,16,17,18,19,20,21]. These muscles are adjacent and/or attached to the ET, and their coordinated contraction dilates the ET lumen, facilitating the clearance and pressure equalizing functions of the ET, thus contributing to the health of the middle ear cavity.

The ET, located deep within the head, is particularly challenging to assess and visualize, even with modern imaging technology, which perhaps explains the relatively few studies that directly compare its shape in groups with known diagnoses. Methodologies for evaluating the shape of this structure range from two-dimensional lateral radiographs [22] to three-dimensional quantification from dry skulls and CT imaging [23,24,25,26,27,28]. Past literature suggests that the angle of the ET changes dramatically from infancy to adulthood and that shape changes in the basicranium and nasopharynx coincide with the timing of peak OM incidence. Those changes include an increase in verticality of the cartilaginous ET, an increase in height and narrowing of the choana, and an increase in basicranial flexion from infancy [27]. These changes are a reflection of the displacements of osseous landmarks created by the attachments of ET soft tissues to bone. The inability of these imaging modalities to discriminate soft tissue differences is the primary challenge for studying the functional cartilaginous ET.

Medical radiographs and computed tomography (CT) scans often fail to capture the cartilaginous ET; even MRI is likely to miss the ET unless the scan is optimized because, under resting conditions, the ET lumen is closed [29]. Smith et al. [29] point out that high-resolution CT and MRI can both be used to assess the ET with relative accuracy, but MRI is preferred because the Ostmann’s fat pad, which runs the length of the ET and has high MRI signal intensity, is particularly useful in identifying the ET [30,31,32,33]. 

The anatomy important for ET function and middle ear health consists of a network of structures located in the anatomical spaces of the head and neck; these spaces include the petrous temporal bone, the parapharyngeal space, the infratemporal fossa, the nasopharynx, and the oropharyngeal isthmus. The shape and orientation of additional structures (e.g., the hard palate) have been shown to play a role in OM risk, and even more structures (e.g., the maxillary sinus and nasal cavity) may play a role in the transmission of pathogens and the development of OM [34,35,36,37,38,39]. Prior studies [16,22,36] have focused on linear and angular measurements to study this interrelated network of structures, but those methods do not account for global shape differences and consequently have the potential to miss more nuanced differences that include multiple structures. Geometric morphometric shape analysis records landmarks as three-dimensional cartesian coordinates. The cartesian coordinates are then plotted and compared to look for global shape differences while minimizing the effects of size, rotation, and translation. 

In the present study, we applied geometric morphometric shape analysis to T1-weighted MRI scans of children with and without a history of OM. Our goal was to study the differences in the cartilaginous ET and the muscles acting on it. We hypothesize that the shape of the basicranium and associated anatomy of the ET would be significantly different between the OM and control groups and that the OM groups would exhibit ETs that were significantly more horizontal.

## 2. Materials and Methods

**Figure 1:**
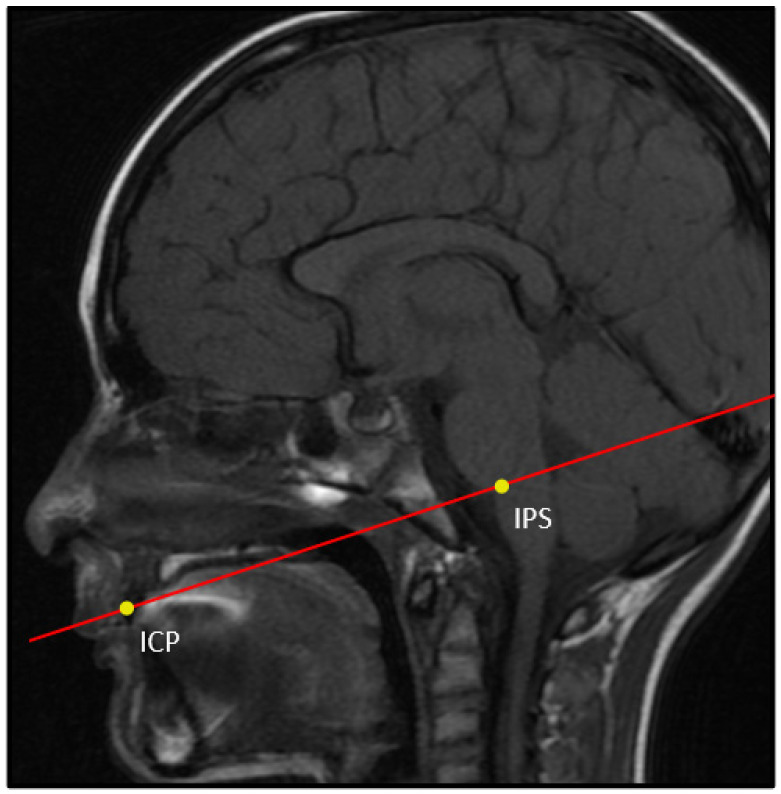
Midsagittal slice of the initial sagittal MRI used to specify the oblique axial scan through the plane of the inferior pontine sulcus and incisive occlusion point. IPS: inferior pontine sulcus (groove between the pons and medulla oblongata); ICP: incisive occlusion point (point of contact between the right and left upper central incisors).

The included children were enrolled in a larger NIH (DC007667)-funded longitudinal study investigating the relationship between age-related changes in ET anatomy and ET function. The children were recruited by advertisements in the Pittsburgh area. The enrolled subjects had an ENT exam every 6 months from 3 to 7.5 years of age. They also had regularly scheduled ET function tests and regularly scheduled measurements of craniofacial anatomy, which included anthropometry, cephalometric measurements, and dental casts. A subset of the enrolled children had a non-sedated MRI between 6 and 7 years of age. A 3 Tesla GE Signa scanner was used to capture the MRI scans. For this analysis, proton density-weighted fast-spin echo scans taken in the oblique axial plane were used (matrix 512 × 512, field of view = 12 cm, 2.0 mm slices). The location for this data scan was obtained by first imaging in the sagittal plane, then using the midsagittal slice to identify the orientation of the oblique axial scan as the plane through the incisive occlusion point and the inferior pontine sulcus (Figure 1). Exclusion criteria for enrollment included any child with (1) a syndrome that predisposed them to OM (e.g., cleft palate), (2) a significant history of orthodontic treatment, (3) a history of ear surgery, excluding ventilation tube insertion, or (4) a child unable to cooperate for testing.

Twenty-three children completed the MRI, and 16 children were included in this analysis. Seven subjects were excluded because the scan area did not include the relevant landmarks. On enrollment in the longitudinal study, the subjects were allocated into one of three groups: control, cOME, and rAOM. The control group had no history of ventilation tubes and did not meet the criteria for the cOME and rAOM groups. The cOME group met at least one of the following criteria: (1) bilateral middle ear effusion for at least 3 consecutive months; (2) unilateral middle ear effusion for at least 6 consecutive months; (3) at least 3 episodes of OM lasting at least 2 months and at least 1 episode within 12 months of enrollment in the study; or (4) ventilation tubes inserted for cOME. The rAOM group met at least one of the following criteria: (1) at least 3 episodes of AOM within one year; (2) at least 5 episodes of AOM by 3 years of age and 2 episodes of AOM within 12 months of enrollment in the study; or (3) ventilation tubes inserted for rAOM. 

Of the 16 children included in the analysis, 5 were in the control group, 3 in the cOME group and 8 in the rAOM group. There were 7 males and 9 females. Race was reported as 1 black, 13 white, and 2 American Indian. The average age at the time of the MRI scan was 6.92 +/−0.33 years.

**Figure 2:**
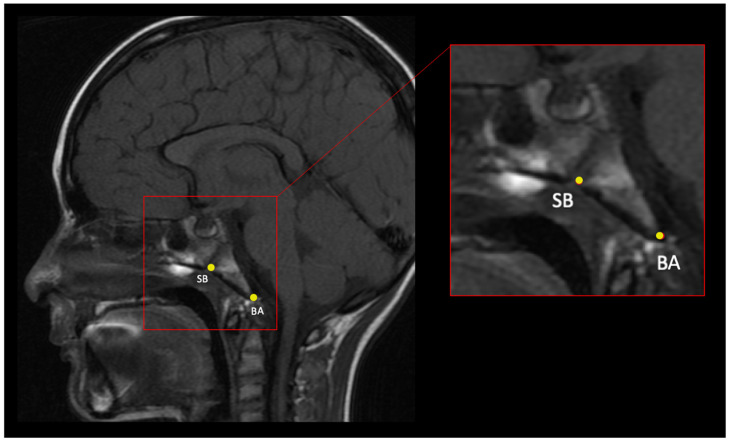
Midsagittal slice from an MRI scan depicting basion (BA) and sphenobasion (SB).

**Figure 3:**
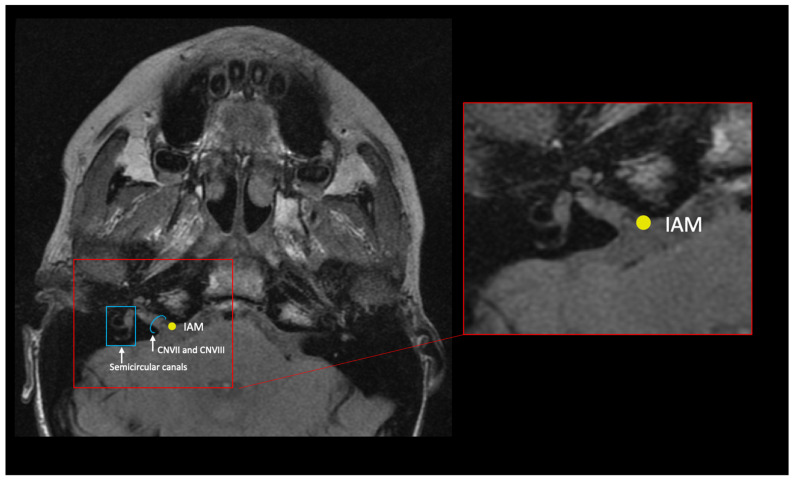
Oblique axial slice from an MRI scan depicting the internal acoustic meatus. CNVII: facial nerve (cranial nerve 7); CNVIII: vestibulocochlear nerve (cranial nerve 8); semicircular canals; IAM: internal acoustic meatus.

**Figure 4:**
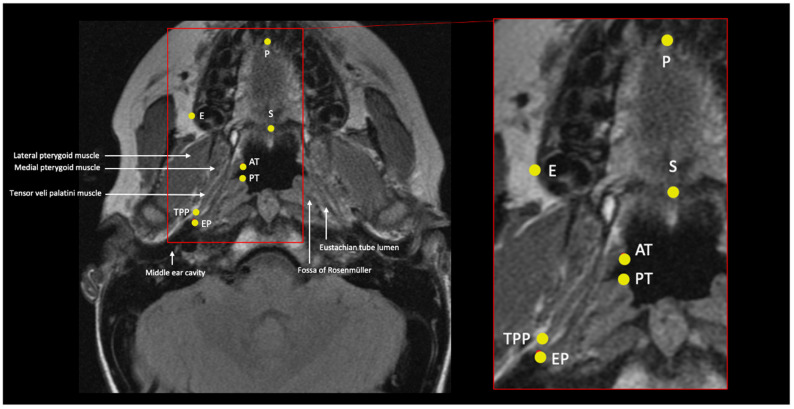
Oblique axial slice from an MRI scan depicting prosthion (P), staphylion(S), ectomolare (E), anterior and posterior torus tubarius (AT and PT), proximal insertion of TVP (TPP), and Eustachian point (EP). This is not the optimal slice for all these landmarks but is included because it demonstrates their relative positions.

**Figure 5:**
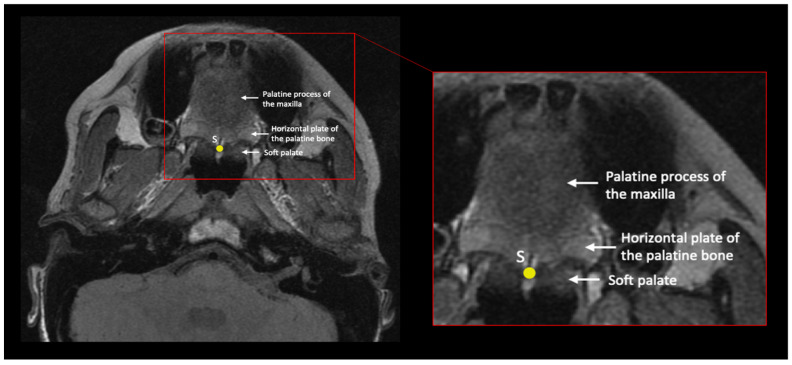
Oblique axial slice from an MRI scan depicting staphylion (S).

Landmarks that captured the shape of the cartilaginous ET, as well as some of the basicranial anatomy, were collected (Table 1 and Figure 2, Figure 3, Figure 4 and Figure 5). Where possible, landmarks were pulled from the literature, many of which are standard craniometric points. Several novel landmarks were established for the present study that describe the soft tissue, including the anterior and posterior torus tubarius and the TVP point. These novel landmarks can only be viewed and analyzed using MRI and specific imaging. Landmarking was done in Checkpoint software on individual slices of the MRI scan and exported to MorphoJ and SPSS for analysis [24,25]. The Procrustes analysis and PCA convert the data (i.e., the three-dimensional Cartesian coordinates) into a shape for each subject. The shapes are then scaled and rotated to find the orientation that produces the least difference among subjects. PCA takes a covariance matrix of the Procrustes aligned coordinates and identifies the axes of greatest variation. Specific shape differences in the cranial base can be isolated and visualized in a scatter plot; further, PC scores can be used to test for significant correlation with an independent variable like OM diagnosis. Each PC explains a different dimension of variance, and variance is a measure of the difference from the mean (i.e., it is the spread of data and more specifically, the average of the squared differences from the mean). The first PC encompasses the greatest proportion of total shape variance, and each consecutive PC represents orthogonal vectors of descending proportions of the variance [40,41]. PCs can be plotted against one another (e.g., PC1 against PC2) or against the centroid size, the calculated size of the shape. Each point in the scatterplot represents the shape of the cranium of a single individual. The data points within the scatterplots can be assigned different colors based on the research group (e.g., control and disease). This is used as a visual representation of the distribution of the research groups. Confidence ellipses can then be used to illustrate if there are shape differences in any of the PCs between the groups. 

A Procrustes ANOVA can be used to test whether shape, as described by the Procrustes distances, is significantly correlated with an independent variable, in this case, OM status. Canonical variate analysis is a complementary analysis that uses the Procrustes distances to identify the shape features that maximize the distance between groups by modifying the data so that within-group variation is isotropic; the variance is equally distributed around the mean. This was particularly useful in a study like this one, with known groupings (OM and control), to test whether the two groups can be significantly separated and identify the shape features that separate them best.

Measurement error was assessed using Procrustes ANOVA in MorphoJ. A second iteration of landmarking was completed on a subset of the sample (*n* = 5) with at least 1 week between trials. Results of the error study indicate that the effect of individual shape remains significant despite intraobserver error associated with landmarking (*p* < 0.01).

## 3. Results

**Figure 6:**
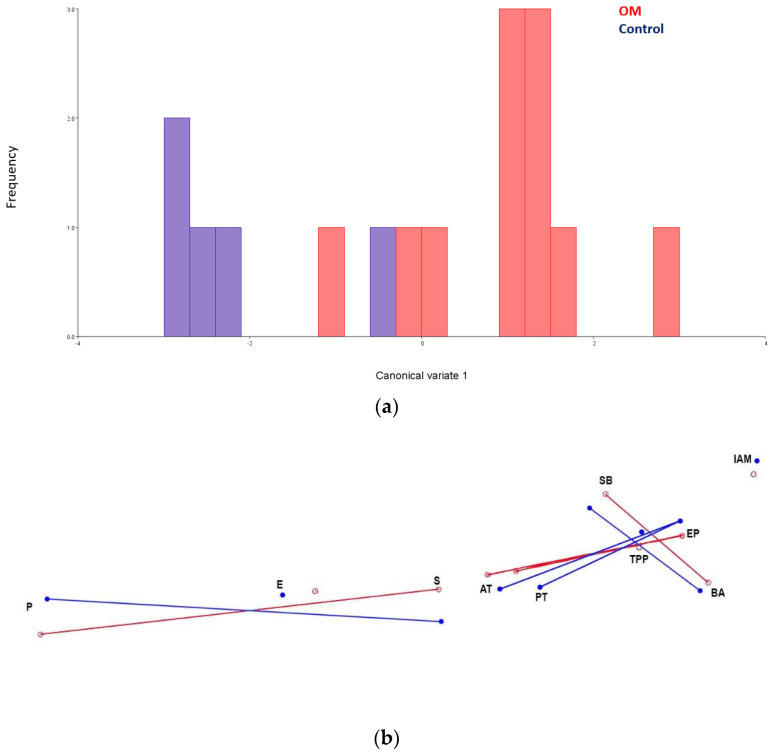
Results of CVA. The OM and control groups separated significantly on CV 1 (**a**). Canonical variate 1 captures a more horizontal ET in the OM group; a lateral view (**b**) of the shape differences illustrates the differences in ET between the OM group (red) and control (blue). Landmarks include internal acoustic meatus (IAM), basion (BA), Eustachian point (EP), tensor veli palatini proximal attachment (TPP), sphenobasion (SB), staphylion (S), ectomalare (E), endoprosthion (P), and anterior and posterior torus tubarius (AT and PT).

The results of the Procrustes ANOVA indicate that the shape and orientation of the ET significantly vary between the OM (combined rAOM, and cOME) and control groups (*p* < 0.01). Results of the CVA support these results, with significant differences found in the Procrustes distances between the OM and control groups (Wilks lambda = 0.226—a Wilk’s lambda close to 0 indicates significant discrimination between groups; Figure 6). A set of ad hoc analyses were completed on a subset of landmarks, excluding those of the palate, because they showed high levels of variability. Palatal variation seems to, at least in part, be swamping out some of the finer variation in the cranial base. In the ad hoc analyses, results remained significant (Wilks lambda = 0.147). 

**Figure 7:**
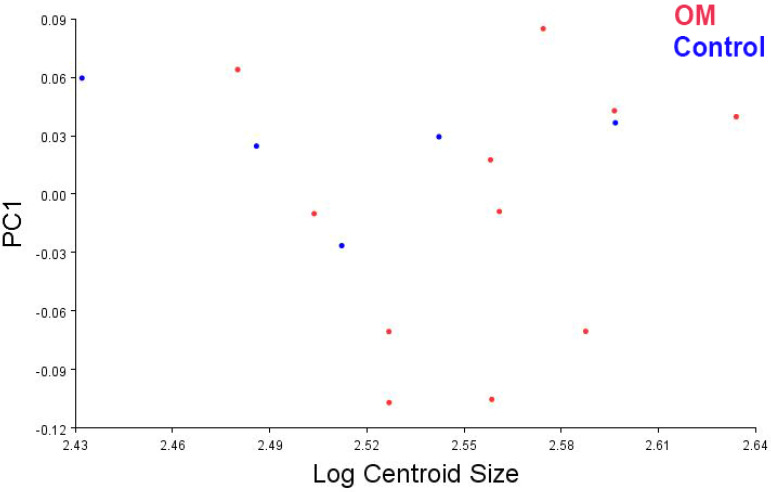
PC plot of PC1 and centroid size. There was no significant relationship between shape and head size.

**Figure 8:**
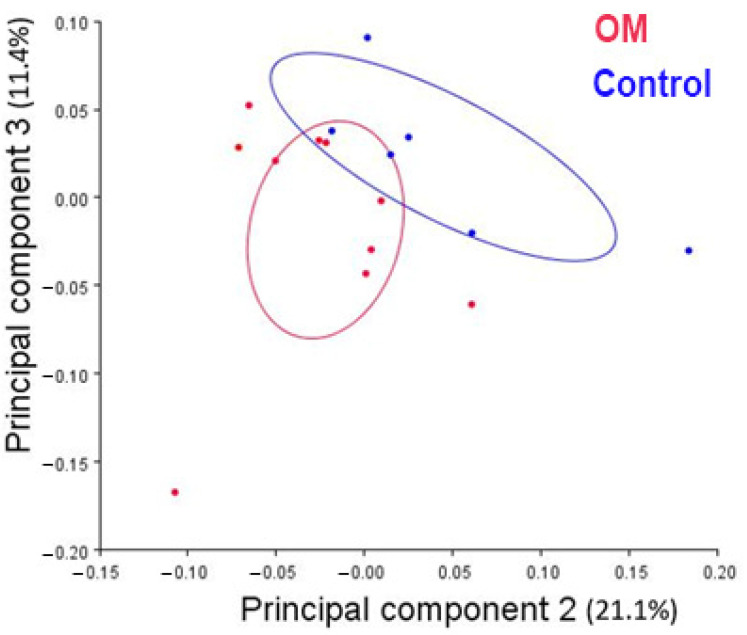
PC plot of PC2 and PC3. OM individuals are illustrated in red, and control in blue. Ellipses illustrate a 95% confidence interval for each group.

**Figure 9:**
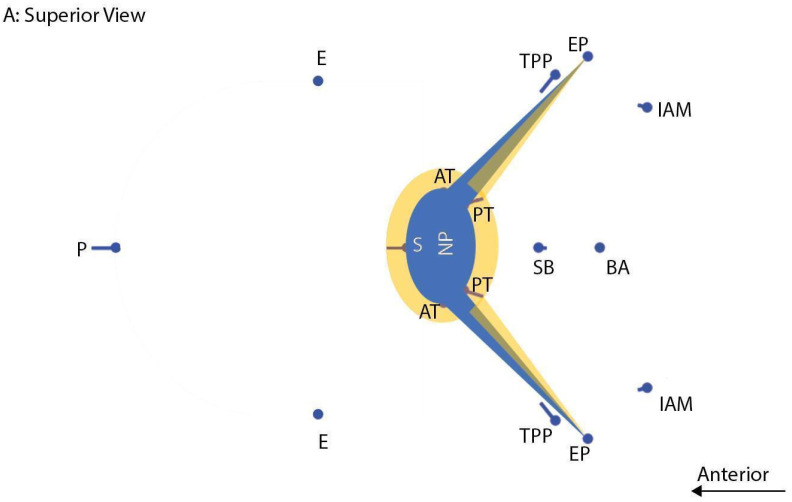

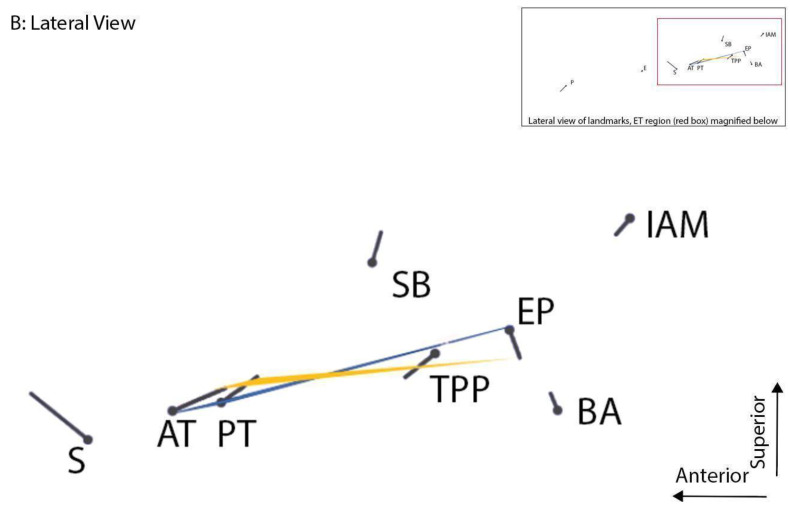
PC3 shape differences. PC 3 captures 11.4% of the variation. Superior (**A**) and lateral (**B**) views of basicranial shape illustrating average (nodes) and high values (lines) on PC 3. The ET angulation and nasopharynx (NP) are illustrated in blue (average) and yellow (OM). Landmarks include internal acoustic meatus (IAM), basion (BA), Eustachian point (EP), tensor veli palatini proximal attachment (TPP), sphenobasion (SB), staphylion (S), ectomalare (E), endoprosthion (P), and anterior and posterior torus tubarius (AT and PT). The node is the average of all individuals, and the vector illustrates variation in the direction of shapes associated with OM diagnosis of PC3.

Results of the PCA show that groups do not separate well on PCs 1 or 2 (Table 2; Figure S1). Principal component 1 (PC1) did not correlate with centroid size (Figure 7; β= −0.32); this result suggests that although PC1 captures most of the variance, it is not associated with head size. Based on the Kruskal–Wallis test, the differences between the groups (control and OM) are best summarized by PC3. PC3 describes a medial and low attachment site of the TVP muscle on the osseous ET, a posteriorly oriented and high torus tubarius, and an anteriorly projected palate in the OM group (rAOM and cOME) compared to the control group (Figure 8 and Figure 9).

## 4. Discussion

Otitis media is extremely common in children under age 2. Many factors contribute to OM susceptibility, including viral type and load, immune system immaturity, life history, and/or craniofacial anatomy, which includes ET shape and orientation. The anatomical and structural factors are at the foundation of OM in infancy, over which the immune system immaturity and infection are overlaid. The peak age of rAOM is around 18 months, but maturing immune defense mechanisms through acquired immunity in response to repeated viral and bacterial infections increase the causal weight of anatomical/structural factors in the etiopathogenesis [9]. In the current study, an enrollment age of 3 years and follow-up until age 7.5 years reduces the impact of infectious and immune etiologies, allowing the role of morphology (i.e., ET shape) on OM incidence to emerge. While rAOM continues to be seen in children older than 3, the prevalence of cOME becomes more dominant after this age [9]. Group assignments may be in error if they are made based on recall or retrospective history. Thus, being part of a prospectively and closely followed cohort employing validated and experienced clinicians, particularly ENT specialists, diminishes the risk of misdiagnosis. The main hypothesis relies on differentiating between children with and without the history of OM. One of the strengths of the current study is having a reliable follow-up and assessment, resulting in an accurate differentiation of study and control groups. 

Many differences between adult and infant ET anatomy exist that may affect susceptibility to OM. Of greatest interest to this study is the relative horizontality of the Eustachian tube, but others are important to note as well, although they could not be tested using the current methodology [42]. Bluestone [42] classifies the pathophysiological effects of ET dysfunction into three categories: impairment of pressure regulation, loss of protective function, and impairment of clearance. The angulation of the ET falls under the category of impairment of pressure regulation due to the ET being too closed. A more horizontal angulation of the ET tube can lead to inefficient action of the TVP and, thus, ineffective opening of the ET. Conversely, ET dysfunction may be caused by the ET being too open (i.e., patulous), which can lead to a loss of the protective function. The cartilage itself is less stiff in the infant and is less supported by a smaller Ostmann’s fat pad, resulting in a floppier ET. Additionally, the ET is relatively short in the infant. These factors also likely play a role in OM susceptibility.

Few studies have directly compared the shape of the craniofacial complex between children with and without a history of OM, at least partially because most children with OM do not require imaging for the diagnosis or treatment of the disease. Using lateral radiographs, Gremba et al. [22] found that the shape of the basicranium in children with rAOM had distinct craniofacial morphologies compared to the control group in their 4-year-old age group. Takasaki et al. [36] found no significant difference in the angle or length of the ET in their sample of children with and without OME. Geometric morphometric shape analysis of MRI scans has several distinct advantages over both linear measurements and X-ray imaging techniques (radiographs and CT scans). First, with MRI scans, soft tissue landmarks may be captured, which is not possible with radiographs or CT scans, and second, MRI scans allow for more accurate placement of some bony landmarks because of the visibility of relative soft tissue. One of the major advantages of this study was to analyze previously unstudied soft-tissue landmarks in relation to OM. Due to MRI scan constraints, novel soft tissue landmarks were limited to the following: the anterior and posterior torus tubarius and tensor veli palatini point. However, the presence of soft tissue structures allowed precise landmarking of the following bony landmarks as well, which would not have been possible with an X-ray or CT scan: Eustachian point and internal acoustic meatus. Therefore, the use of MRI scans (i.e., visualization of soft tissue structures) was essential in placing landmarks for 5/10 landmarks, and if we exclude the landmarks of the maxilla and cranial base, then it was essential for 5/6 ET and TVP-specific landmarks. 

Geometric morphometric analysis offers its own advantages over linear measurements because it reduces differences due to size and tests the differences between groups based on the relative location of landmarks to one another. Therefore, the plane in which the data was collected does not matter. Pagano et al. [25] found that the length and shape of the cartilaginous ET, as represented by bony proxies in dry skulls, changes dynamically throughout infancy and early childhood and correlates with peak incidences of OM. OM is much less common by the age of six but can and does occur into later childhood and adulthood [29]. It is possible that some of the same variables that go into OM susceptibility in infancy persist [43]. Relatively few studies exist that directly compare children with known medical histories, and even fewer take into account soft tissue and/or three-dimensional relationships. The results of this study indicate there are significant differences in anatomical relationships between groups with and without a history of OM. 

Previous studies have found that the TVP muscles are necessary for the opening of the ET lumen, and by extension, the function of these muscles may be an important protection of the middle ear that may help prevent OM [11,19,34]. Sapci et al. [44] is one of the few studies that directly compared TVP muscle electromyography in adult subjects with or without OM, and they found no difference in motor unit potential or duration of the TVP. While the present study did find differences in the position of the proximal insertion of the TVP on the cranial base, the TVP landmarks varied with the middle ear landmark Eustachian point. So, while the TVP was found to be oriented more inferiorly in the OM group, it did not vary relative to the positioning of the middle ear in the PCs, which captured a significant amount of the variation. Regardless, it is possible that the angle of the TVP relative to the hamulus of the pterygoid plates (the fulcrum around which the TVP is set) is less acute and thus may be suboptimal in the OM group. One thing that could not be tested using the method presented here is the insertion ratio or the relative length of the insertion of the TVP to the total length of the ET [45]. It may be that a lower posterior boundary of the TVP attachment would mean a lower anterior boundary as well, but it is certainly possible that the thickness of the muscle varies, not the total position.

It is interesting that the variation at the palate had to be removed in some analyses because it was “swamping out” the fine-grained differences at the ET. The relationship between OM and palate shape is frequently discussed in clinical research, as exemplified by the extensive literature concerning cleft palate and OM (e.g., [20,34,35,44,46]). A cleft palate leads to many anatomical and structural differences in the craniofacial complex that may lead to OM, including differences in TVP size as measured on MRI in adults with and without a cleft palate [47]. The shape of the palate certainly seems to vary between patients with and without OM in this study, though it was not specifically isolated and analyzed. The palate shape is integrated with the shape of the oral cavity and dentition—especially the mixed dentition, which characterizes this age group. Further, the shape of the palate reflects the general width of the face. The oral vestibule and palate are continuous with the nasopharynx posteriorly; thus, an anteriorly projected palate could be associated with a long (anteroposterior) nasopharynx. A large nasopharynx would have implications for the position of the hamulus of the pterygoid plates. The presented results provide a basis upon which future analyses of the palate shape in OM children can be based.

## 5. Conclusions

This study contributes to the growing literature on imaging and shape analysis of the ET. This is the first attempt that the authors could find at using MRI data to perform geometric morphometric shape analysis on the ET in any age group. In the present study, we found evidence that the shape of the Eustachian tube varies significantly between these groups of children with and without OM.

## 6. Limitations and Future Directions

It is important to note our limitations. The sample size is small, and a larger sample would increase the power of the statistical analyses. However, despite the small sample size, the authors found significant differences between cohorts and established novel protocols for the collection of soft tissue landmarks in the ET that can be used in future studies. The soft tissue landmarks presented here open up the types of hypotheses that can be tested using three-dimensional geometric morphometrics of the middle ear and its pathologies.

While the MRI scans capture an enormous amount of anatomy, the slice thicknesses and window sizes limit the types of landmarks collected. Certain biologically significant landmarks could not be collected in the present study due to the window size of the original scans, including landmarks around the paranasal sinus drainage points, nasal, and nasopharyngeal landmarks. Further, placing landmarks on individual slices did not allow for the use of semilandmarks that extended beyond a single plane. This can be addressed in the future by using this pilot protocol and adjusting scan settings to widen the scan windows and narrow the slice thicknesses.

In the future, this method should be tested with a larger sample and other age ranges, particularly in the older ages that have been historically under-evaluated in the literature. With a larger sample size, the authors will be able to test for potentially confounding or informative variables like sex, population affinity, and life history. Further, digital isolation of the ET and the paratubal muscles will allow for a wider range of statistical analyses.

## Figures and Tables

**Table 1:** Landmarks included in the present study.

Bone Landmark	Abbr.	Location	Definition	Description	Figure
Sphenobasion	SB	Midline	Midline of spheno-occipital synchondrosis	In a midsagittal plane, identify the clivus and place the landmark on the superior border of the clivus on the external surface in between the sphenoid and occipital bones; this is a cartilaginous plate in children.	Figure 2
Basion (exo-basion)	BA	Midline	Midline of anterior foramen magnum	In a midsagittal plane, identify the most inferior–posterior point on the clivus.	Figure 2
Staphylion	S	Midline	Posterior maxillary spine	In the oblique axial plane, identify the slice that contains the maxillary palatine process and palatine bones; these form the hard palate. Place the landmark on the most posteromedial point of the palatine palate.	Figure 4 and Figure 5
Prosthion (endoprosthion)	P	Midline	Midline of the anterior palate	In the oblique axial plane, identify the slice that contains the cementoenamel junction of the upper central incisors (the cross-section of the incisors will be oval). Place the landmark on the lingual surface of the palate between the right and left upper central incisors at the level of the cementoenamel junction.	Figure 4
Internal acoustic meatus	IAM	Right and left	Internal opening of the petrous temporal bone	In the oblique axial plane, identify the facial and vestibulocochlear nerves emerging from the brainstem and follow the nerves medially. Locate the point of greatest curvature at the anterior entry to the IAM.	Figure 3
Eustachian point	EP	Right and left	Junction of bony and cartilaginous ET	In the oblique axial plane, identify the external acoustic meatus, and follow the space into the middle ear cavity, and place the landmark at the most anteromedial opening.	Figure 4
Proximal tensor veli palatini attachment point	TPP	Right and left	Origin of the TVP muscle at the skull base	In the oblique axial plane, identify the TVP muscle fibers and follow them proximally (toward the cranial base), and place the landmark at the most proximal insertion.	Figure 4
Anterior torus tubarius	AT	Right and left	Anterior border of the distal ET	In the oblique axial plane, identify anterior and posterior medial ends of the nasopharyngeal orifice of the ET.	Figure 4
Posterior torus tubarius	PT	Right and left	Posterior border of the distal ET		Figure 4
Ectomolare	E	Right and left	Most lateral point on the exterior surface of the alveolus	In the oblique axial plane, identify the slice with the widest alveolus and palate. Place the landmark on the external surface of the alveolus at the widest point.	Figure 4

**Table 2:** Description of shape variation captured in PCs 1–3. PC 3 is the only PC that significantly correlated with OM diagnosis.

PC	% Variance	Total Variance	Cranial Base	Eustachian	Torus Tubarius
1	29.8	29.8	Wide (medial/lateral)	Lateral	Anterior
2	21.1	50.9	Tall (superior/inferior)	Inferior	High
3	11.4	62.3	Short	Inferior	Posterior, superior

## Data Availability

Data are available upon request.

## Supplementary Materials

The following supporting information can be downloaded at: https://www.mdpi.com/article/10.3390/bioengineering10101115/s1.
