# Supplementary material for: Using Geometric Morphometric Analysis of Magnetic Resonance Imaging to Assess the Anatomy of the Eustachian Tube in Children with and without Otitis Media
Source: Bioengineering (Basel). 2023 Sep 23;10(10):1115. doi: 10.3390/bioengineering10101115 (PMC10604907; doi:10.3390/bioengineering10101115)
Supplement: Supplementary file 1 [file bioengineering-10-01115-s001.zip › bioengineering-2553196-supplementary.pdf]

Supplement 1: PC plot of PCs 1 and 2

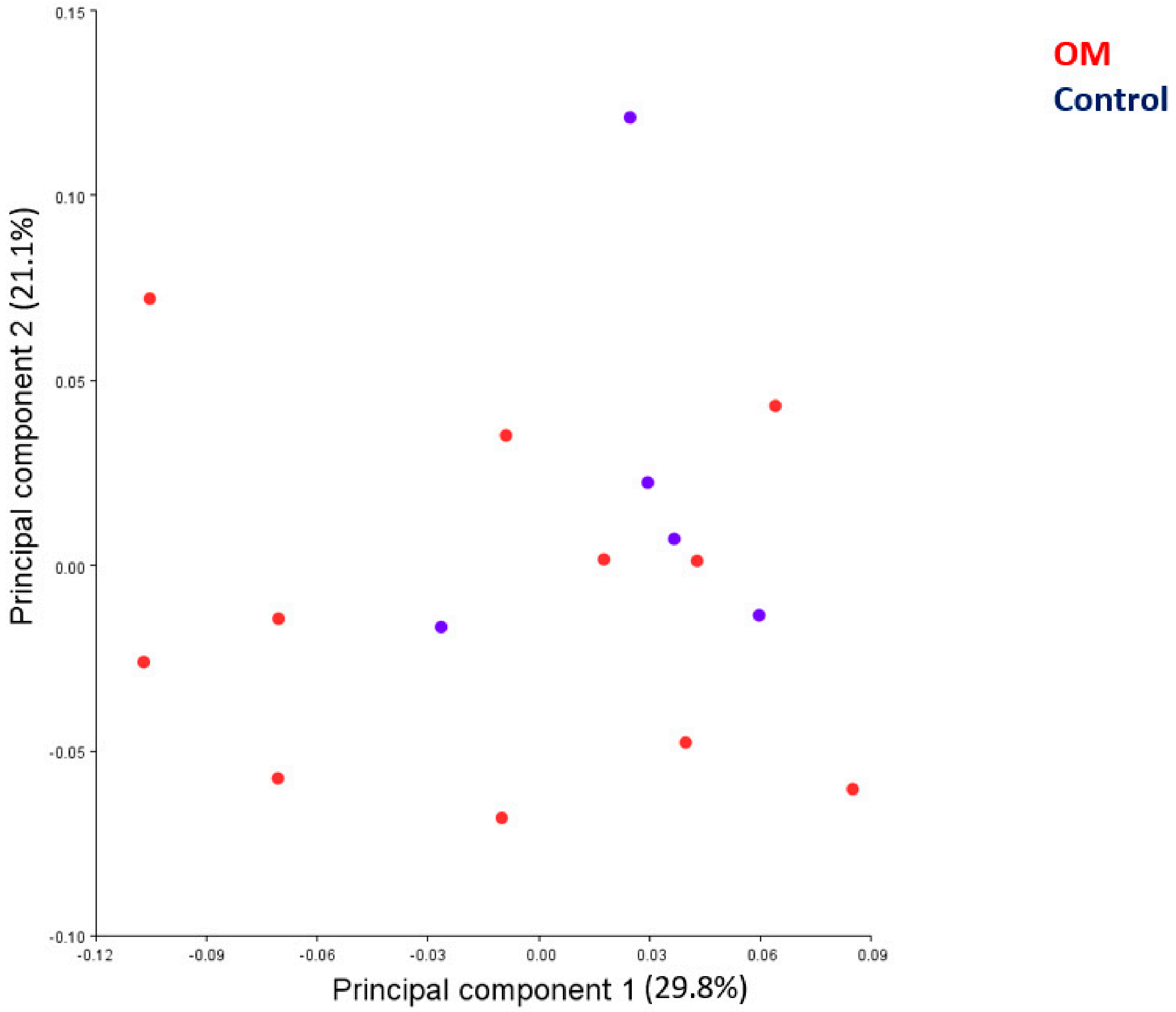

Supplement 2: Principal components coefficients

Principal Component Coefficients

	PC1	PC2	PC3	PC4	PC5	PC6	PC7	PC8	PC9	PC10	PC11	PC12	PC13	PC14	PC15
x1	0.060918	-0.060656	0.083884	-0.241425	-0.044764	-0.032338	-0.207926	-0.383970	-0.038223	0.184722	0.031293	-0.259076	-0.571507	-0.310303	0.151693
y1	0.000000	0.000000	0.000000	0.000000	0.000000	0.000000	0.000000	0.000000	0.000000	0.000000	0.000000	0.000000	0.000000	0.000000	0.000000
z1	-0.009218	0.197212	0.153975	-0.109125	0.124901	0.121315	0.104089	-0.161870	-0.368419	-0.149977	-0.072701	0.350224	-0.015448	0.067855	0.032409
x2	0.027826	-0.066404	-0.014952	-0.165603	-0.161139	-0.105639	-0.160376	-0.077303	0.176260	-0.225538	0.132355	0.119142	0.078866	-0.137028	0.180389
y2	0.000000	0.000000	0.000000	0.000000	0.000000	0.000000	0.000000	0.000000	0.000000	0.000000	0.000000	0.000000	0.000000	0.000000	0.000000
z2	0.019055	0.167827	0.099596	-0.042951	0.041574	-0.155648	-0.037652	0.036690	-0.022910	0.507892	-0.389411	-0.159437	-0.189508	0.298756	0.151687
x3	-0.266246	0.233922	-0.254112	0.139540	0.416420	0.026553	-0.188161	-0.006060	0.355619	0.024065	0.280031	-0.050814	-0.024779	0.324597	0.134208
y3	0.000000	0.000000	0.000000	0.000000	0.000000	0.000000	0.000000	0.000000	0.000000	0.000000	0.000000	0.000000	0.000000	0.000000	0.000000
z3	0.289102	-0.125492	0.323745	-0.072221	-0.273085	0.105795	-0.138524	-0.180339	0.029424	0.185284	0.338588	0.213474	0.180001	0.442029	0.223255
x4	-0.142622	0.027888	-0.308777	0.195633	-0.206157	-0.094931	0.241500	-0.031940	-0.460423	0.056073	0.303988	0.091744	-0.156746	-0.005885	0.193976
y4	0.000000	0.000000	0.000000	0.000000	0.000000	0.000000	0.000000	0.000000	0.000000	0.000000	0.000000	0.000000	0.000000	0.000000	0.000000
z4	-0.315788	-0.087269	-0.191186	-0.169160	0.061877	0.204235	-0.192653	0.073535	-0.148123	0.258956	-0.141604	-0.069545	0.283442	-0.139032	0.060870
x5	-0.100278	0.020027	-0.098009	-0.258085	0.046110	-0.192930	0.044995	0.012902	0.043482	-0.016381	0.093920	0.171516	-0.050252	0.168886	-0.147535
y5	-0.055950	-0.110173	0.022475	0.137064	0.195352	0.053582	-0.268489	0.002860	-0.309224	-0.151144	-0.081806	0.199148	-0.155371	0.070428	-0.001425
z5	-0.011730	-0.001014	-0.072496	-0.173171	0.009303	0.201687	-0.105402	0.409970	-0.033649	0.030999	0.160354	-0.037447	-0.057793	-0.143071	0.061251
x6	-0.000360	0.187235	0.022593	0.009281	-0.128740	-0.045951	0.218293	0.135424	0.015734	0.123699	-0.190945	0.207467	0.132024	-0.134348	0.141617
y6	0.120754	0.137194	0.024188	0.168508	0.261009	0.058351	0.216253	-0.063942	-0.157936	0.169492	0.155646	-0.154418	0.035590	0.018225	-0.054887
z6	-0.025034	-0.289549	-0.166234	0.133368	0.173563	-0.103945	0.062116	-0.271476	0.148800	0.102947	-0.089091	0.168630	0.134832	-0.183129	0.047496
x7	0.069683	-0.042938	-0.193911	0.139158	-0.232742	0.106766	-0.052558	0.002373	-0.018095	-0.013971	-0.223310	-0.075939	-0.049423	0.156683	-0.250609
y7	0.161894	0.022987	-0.226806	0.213366	-0.155018	0.294364	-0.029674	-0.072921	0.059333	0.016029	0.086218	-0.032873	-0.001351	-0.032496	0.166022
z7	-0.058463	-0.173683	-0.092426	-0.053661	-0.121445	-0.061217	0.038053	-0.053412	-0.113061	-0.074974	0.038455	-0.207596	0.090608	0.154439	-0.228080
x8	-0.119339	-0.112386	0.317578	0.095367	0.053794	0.199518	0.075613	0.018494	0.080923	0.080119	0.083939	-0.054569	0.033222	-0.031008	-0.174914
y8	-0.069502	0.006768	0.113776	0.204725	-0.109241	-0.191244	-0.176972	0.075003	-0.049454	0.255528	0.220725	0.111568	0.019667	-0.196780	-0.264201
z8	-0.097500	0.277040	0.060792	0.103534	-0.061282	0.000003	-0.039592	-0.109159	0.069520	-0.174988	-0.054238	-0.091872	0.001671	-0.006564	-0.037816
x9	-0.115862	-0.213081	0.229833	0.079982	0.067216	0.102613	0.103820	0.052117	-0.039209	-0.155441	-0.082172	-0.144293	0.044168	-0.002063	0.177113
y9	-0.062652	-0.064960	0.076360	0.284802	-0.075277	-0.265690	-0.200048	0.186392	0.002490	-0.032623	-0.073321	-0.048503	0.014517	0.084656	0.220846
z9	-0.056637	0.262498	0.097393	0.100192	-0.078577	-0.047221	-0.036795	-0.090068	0.056717	-0.188852	0.039849	-0.037954	-0.024676	-0.155427	-0.048668
x10	0.426218	0.093768	-0.031106	-0.029775	0.192183	-0.066839	-0.232680	0.028326	-0.099452	-0.037686	-0.055265	-0.054681	0.227344	-0.093841	-0.075806
y10	-0.000985	-0.022818	-0.015130	-0.086160	0.003319	-0.252702	0.071467	-0.015979	-0.166864	-0.077779	0.118468	-0.293040	0.195049	-0.017028	0.146575
z10	0.257788	-0.151430	-0.020094	0.086467	0.100805	-0.127155	0.213989	0.230137	0.126688	-0.096209	0.037235	0.038882	-0.273885	-0.001052	-0.028294
x11	-0.100278	0.020027	-0.098009	-0.258085	0.046110	-0.192930	0.044995	0.012902	0.043482	-0.016381	0.093920	0.171516	-0.050252	0.168886	-0.147535
y11	0.055950	0.110173	-0.022475	-0.137064	-0.195352	-0.053582	0.268489	-0.002860	0.309224	0.151144	0.081806	-0.199148	0.155371	-0.070428	0.001425
z11	-0.011730	-0.001014	-0.072496	-0.173171	0.009303	0.201687	-0.105402	0.409970	-0.033649	0.030999	0.160354	-0.037447	-0.057793	-0.143071	0.061251
x12	-0.000360	0.187235	0.022593	0.009281	-0.128740	-0.045951	0.218293	0.135424	0.015734	0.123699	-0.190945	0.207467	0.132024	-0.134348	0.141617
y12	-0.120754	-0.137194	-0.024188	-0.168508	-0.261009	-0.058351	-0.216253	0.063942	0.157936	-0.169492	-0.155646	0.154418	-0.035590	-0.018225	0.054887
z12	-0.025034	-0.289549	-0.166234	0.133368	0.173563	-0.103945	0.062116	-0.271476	0.148800	0.102947	-0.089091	0.168630	0.134832	-0.183129	0.047496
x13	0.069683	-0.042938	-0.193911	0.139158	-0.232742	0.106766	-0.052558	0.002373	-0.018095	-0.013971	-0.223310	-0.075939	-0.049423	0.156683	-0.250609
y13	-0.161894	-0.022987	0.226806	-0.213366	0.155018	-0.294364	0.029674	0.072921	-0.059333	-0.016029	-0.086218	0.032873	0.001351	0.032496	-0.166022
z13	-0.058463	-0.173683	-0.092426	-0.053661	-0.121445	-0.061217	0.038053	-0.053412	-0.113061	-0.074974	0.038455	-0.207596	0.090608	0.154439	-0.228080
x14	-0.119339	-0.112386	0.317578	0.095367	0.053794	0.199518	0.075613	0.018494	0.080923	0.080119	0.083939	-0.054569	0.033222	-0.031008	-0.174914
y14	0.069502	-0.006768	-0.113776	-0.204725	0.109241	0.191244	0.176972	-0.075003	0.049454	-0.255528	-0.220725	-0.111568	-0.019667	0.196780	0.264201
z14	-0.097500	0.277040	0.060792	0.103534	-0.061282	0.000003	-0.039592	-0.109159	0.069520	-0.174988	-0.054238	-0.091872	0.001671	-0.006564	-0.037816
x15	-0.115862	-0.213081	0.229833	0.079982	0.067216	0.102613	0.103820	0.052117	-0.039209	-0.155441	-0.082172	-0.144293	0.044168	-0.002063	0.177113
y15	0.062652	0.064960	-0.076360	-0.284802	0.075277	0.265690	0.200048	-0.186392	-0.002490	0.032623	0.073321	0.048503	-0.014517	-0.084656	-0.220846
z15	-0.056637	0.262498	0.097393	0.100192	-0.078577	-0.047221	-0.036795	-0.090068	0.056717	-0.188852	0.039849	-0.037954	-0.024676	-0.155427	-0.048668
x16	0.426218	0.093768	-0.031106	-0.029775	0.192183	-0.066839	-0.232680	0.028326	-0.099452	-0.037686	-0.055265	-0.054681	0.227344	-0.093841	-0.075806
y16	0.000985	0.022818	0.015130	0.086160	-0.003319	0.252702	-0.071467	0.015979	0.166864	0.077779	-0.118468	0.293040	-0.195049	0.017028	-0.146575
z16	0.257788	-0.151430	-0.020094	0.086467	0.100805	-0.127155	0.213989	0.230137	0.126688	-0.096209	0.037235	0.038882	-0.273885	-0.001052	-0.028294
